# Incidence of Guillain-Barré Syndrome (GBS) in Latin America and the Caribbean before and during the 2015–2016 Zika virus epidemic: A systematic review and meta-analysis

**DOI:** 10.1371/journal.pntd.0007622

**Published:** 2019-08-26

**Authors:** Ariadna Capasso, Danielle C. Ompad, Dorice L. Vieira, Annelies Wilder-Smith, Yesim Tozan

**Affiliations:** 1 NYU College of Global Public Health, New York University, New York, New York, United States of America; 2 New York University Health Sciences Library, NYU School of Medicine, NYU Langone Medical Center, New York, New York, United States of America; 3 Department of Disease Control, London School of Hygiene and Tropical Medicine, London, United Kingdom; 4 Department of Global Health and Epidemiology, University of Umea, Umea, Sweden; University of Queensland, AUSTRALIA

## Abstract

**Background:**

A severe neurological disorder, Guillain-Barré syndrome (GBS) is the leading cause of acute flaccid paralysis. Enhanced surveillance of GBS in Latin America and the Caribbean (LAC) following the 2015–2016 Zika virus (ZIKV) epidemic presents an opportunity to estimate, for the first time, the regional incidence of GBS.

**Methods and findings:**

For this systematic review and meta-analysis, we searched nine scientific databases and grey literature from January 1, 1980 to October 1, 2018. Sources with primary data on incident GBS cases in LAC within a well-defined population and timeframe, published in English, Spanish, Portuguese, or French, were included. We calculated the annual GBS incidence rates (IRs) and 95% confidence intervals (CIs) for each source based on published data. Following an assessment of heterogeneity, we used random-effects meta-analysis to calculate the pooled annual IR of GBS. The study is registered with PROSPERO, number CRD42018086659. Of the 6568 initial citation hits, 31 were eligible for inclusion. Background annual GBS IRs in Latin America ranged from 0.40 in Brazil to 2.12/100,000 in Chile. The pooled annual IR in the Caribbean was 1.64 (95% CI 1.29–2.12, *I*^*2*^<0.01, p = 0.44). During the ZIKV epidemic, GBS IRs ranged from 0.62 in Mexico to 9.35/100,000 in Martinique. GBS increased 2.6 (95% CI 2.3–2.9) times during ZIKV and 1.9 (95% CI 1.1–3.4) times during chikungunya outbreaks over background rates. A limitation of this review is that the studies included employed different methodologies to find and ascertain cases of GBS, which could contribute to IR heterogeneity. In addition, it is important to consider that data on GBS are lacking for many countries in the region.

**Conclusions:**

Background IRs of GBS appear to peak during arboviral disease outbreaks. The current review contributes to an understanding of the epidemiology of GBS in the LAC region, which can inform healthcare system planning and preparedness, particularly during arboviral epidemics.

**Trial registration:**

Registered with PROSPERO: CRD42018086659.

## Author summary

A severe neurological disorder, Guillain-Barré syndrome (GBS) is the leading cause of acute flaccid paralysis. This is the first systematic review on GBS incidence in Latin America and the Caribbean before and during arboviral disease outbreaks. There is a large sub-regional and annual fluctuation in the incidence of GBS. Background annual GBS incidence rates (IRs) in Latin America ranged from 0.40 in Brazil to 2.12/100,000 in Chile. The pooled annual IR in the Caribbean was 1.64 (95% CI 1.29–2.12, *I*^*2*^<0.01, p = 0.44). During the ZIKV epidemic, GBS IRs ranged from 0.62 in Mexico to 9.35/100,000 in Martinique. GBS increased 2.6 times during ZIKV and 1.9 times during chikungunya outbreaks over background rates. GBS is a costly disease, which can result in long-term disability and high mortality rates in resource constrained healthcare settings. Because GBS can be triggered by arboviral infections, baseline incidence of GBS is critical for detecting neglected tropical disease outbreaks. The current review contributes to an understanding of the epidemiology of GBS in the LAC region, which can inform healthcare system planning and preparedness.

## Introduction

A rare but severe autoimmune neuropathy, Guillain-Barré syndrome (GBS) is the most common type of acute flaccid paralysis (AFP) [1]. Often preceded by infections such as *Campylobacter jejuni*, about 25% of patients require mechanical ventilation [2]. Prognosis varies greatly based on GBS type and urgent care availability [2–5]. Many patients report residual deficits, including pain, limited mobility, and fatigue, years after disease onset [4, 6–8]. Mortality rates range from 3−7% [3, 9], although they can be higher in settings with limited access to intensive care [10, 11]. In 2008, the total annual cost of GBS in the U.S. alone was estimated at $1.7 billion (95% CI $1.6 to 1.9 billion) [12].

The median global incidence rate (IR) of GBS was estimated at 1.10 per 100,000 person-years (range, 0.81–1.89) [13]. However, this estimate was based on data from studies conducted in Europe and North America [13]. Worldwide, there are large variations in the incidence of GBS, ranging from 0.38 (95% CI 0.25–0.56) to 2.53 (95% CI 1.87–3.56) per 100,000, with most studies reporting annual IRs between 1.1 and 1.8 per 100,000 [14]. Prior to the 2015–2016 Zika virus (ZIKV) epidemic in Latin America and the Caribbean (LAC), there were few published studies on the incidence of GBS in the region, with an exception among children. As part of polio eradication efforts, AFP in children under 15 years of age has been a notifiable event in all LAC countries since the 1980s [15–17]. Using polio eradication surveillance data, in 2010, Landaverde et al. estimated 0.82 cases of GBS per 100,000 among children under 15 years of age (range, 0.72–0.90) [18].

ZIKV is an enveloped positive-strand RNA member of the *Flavivirus* genus in the Flaviviridae family. Other flaviviruses include dengue, yellow fever, West Nile virus and Japanese encephalitis virus, many of which are associated with neurological disease. Like these viruses, ZIKV is principally transmitted by a mosquito bite and is thus described as an arthropod-borne virus or ‘arbovirus’. Its primary vector is the *Aedes aegypti* mosquito, which transmits the virus between humans and is widespread in tropical regions [19].

ZIKV is known to be neurotropic; infection halts proliferation of neural progenitor cells and may induce cell death, leading to ZIKV-related microcephaly [20]. Beyond congenital Zika syndrome, direct viral invasion as well as a parainfective or postinfective autoimmune response may contribute to GBS pathogenesis [21, 22]. An association between GBS and ZIKV was first established in a case-control study in French Polynesia [23]. During the 2015–2016 ZIKV epidemic, many countries in LAC reported increases in GBS cases, particularly in the beginning of 2016 [24, 25]. In 2016, the World Health Organization (WHO) concluded that ZIKV infection was a plausible trigger for GBS [26]. The chikungunya (CHIKV) [27, 28] and dengue (DENV) viruses [29], two other arboviruses that are endemic in parts of the LAC region, have also been investigated as possible GBS antecedent infectious agents.

Enhanced GBS surveillance [30] and increased research present a unique opportunity to assess the background IR of GBS in the LAC region in the aftermath of recent arboviral epidemics. This review aims to assess the background population-wide incidence of GBS in Latin America and the Caribbean through a synthesis of observational studies. For the purposes of this study, “background incidence” refers to the rates reported during time periods with no arboviral epidemic. To our knowledge this is the first systematic review on GBS in this region. A secondary aim of this review is to ascertain the incidence of GBS during arboviral disease outbreaks.

## Methods

### Search strategy and selection criteria

Our protocol followed the Preferred Reporting Items for Systematic Reviews and Meta-Analyses protocols (PRISMA-P) guidelines and the Meta-analyses of Observational Studies in Epidemiology (MOOSE) Checklist (see S1 and S2 Files) [31]. We developed the search word criteria by reviewing PubMed MeSH and Embase Emtree subject headings and keywords. We searched nine electronic databases. Our full search terms are provided in the appendix. We conducted a grey literature search on OpenGrey and OAIster and hand-searched relevant journals and the bulletins of ministries of health cited in bibliographic references of the articles selected for full-text review (see S3 File). We also reviewed the references of all included articles. Inclusion criteria were peer-reviewed and government publications that presented primary data on incident GBS cases in LAC within a well-defined population and timeframe and that were published in English, Spanish, Portuguese, or French between January 1, 1980 to October 1, 2018. The search timeframe was decided upon to update the worldwide review by McGrogan [14] from 1980 to 2008. Review papers were excluded. After removing duplicates, two authors (AC, DLV) independently screened articles by title and abstract based on the inclusion criteria and agreed on those for full-text review. A standard extraction form was developed and tested for reliability. Disagreements were resolved by these two reviewers.

### Data analysis

One author (AC) extracted the following items from the included articles onto the extraction form: study design, country, region, data collection year(s), population size, age, sex, GBS type, GBS diagnostic criteria, incident cases, statistical measures, and circulating arboviral diseases (ZIKV, DENV, or CHIKV). Three other authors (DLV, DCO, YT) extracted data from 30% of the records for quality control.

All data were standardized to annual mean IR by dividing the number of GBS cases reported by the number of weeks of data capture and multiplying the result by 52 weeks. The result was entered as annual GBS cases into the software program, and together with the base population, was used to calculate the annual IR per 100,000 persons. When necessary, we attempted to contact the authors to obtain clarifying information. Three authors (AC, DCO, YT) assessed studies for risk of bias using a tool developed for prevalence studies [32]. To reduce publication bias [33], we searched the grey literature, institutional websites, and conference abstracts. The study protocol was registered with PROSPERO (CRD42018086659, available at https://www.crd.york.ac.uk/prospero/display_record.php?RecordID=86659).

We performed the meta-analysis using the *metaprop* command in Stata version 15 (College Station, Texas, USA). Annualized GBS cases and population sizes were entered for every selected study. Given high heterogeneity, we performed sub-group analyses by: geography (Latin America versus the Caribbean; Southern Cone, Central and North America, and the Caribbean); time (before and during an epidemic outbreak); population (all and under 15 years of age); and case ascertainment (administrative data using ICD codes only and medical record review). Double arcsine transformation and random-effects models were used to calculate pooled IR estimates of GBS [34].

## Results

**Fig 1:**
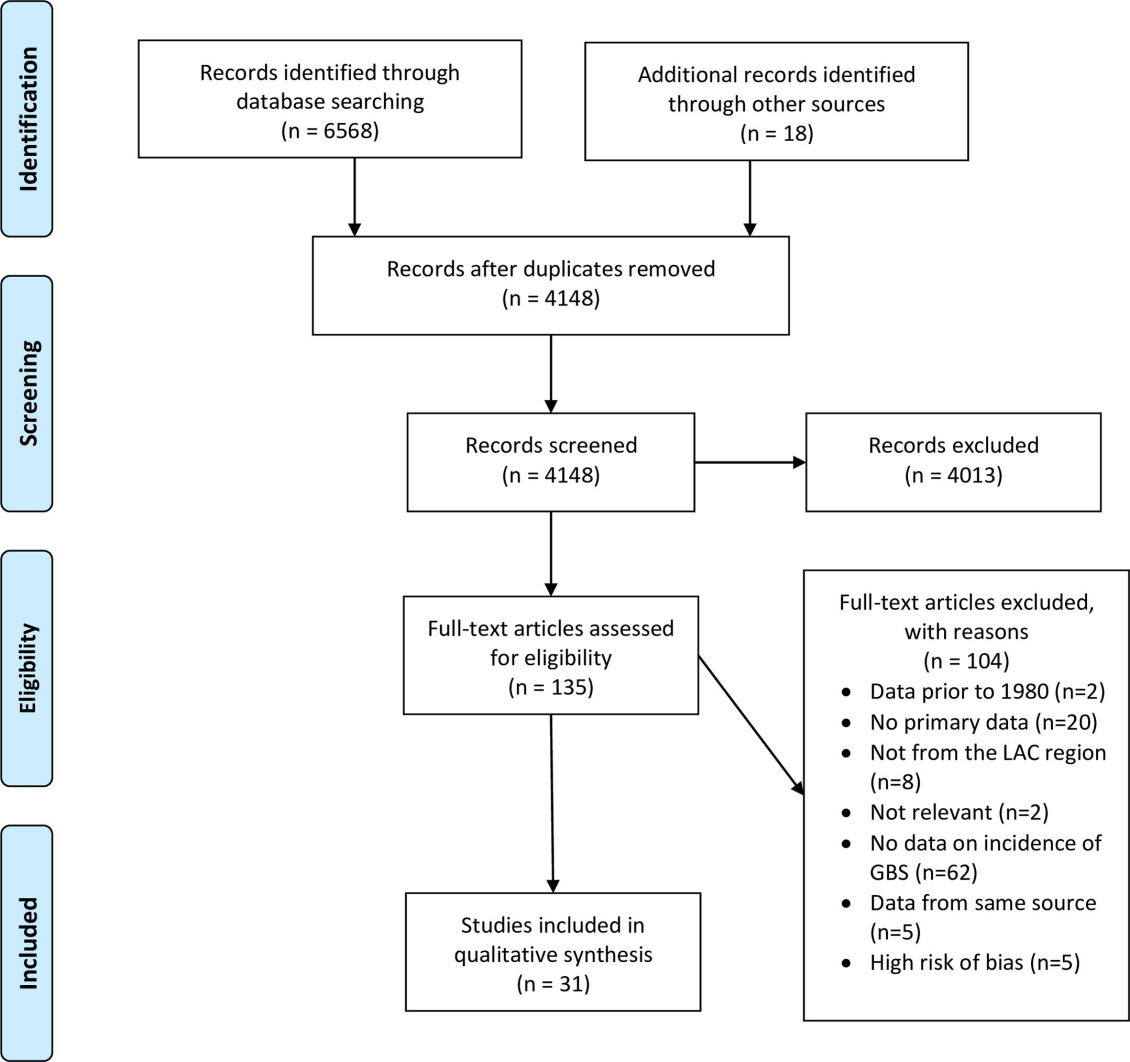
PRISMA flow chart for a systematic review and meta-analysis of Guillain-Barré Syndrome (GBS) in Latin America and the Caribbean before and during the 2015–2016 Zika virus epidemic.

The database search identified 6568 citations. An additional 18 citations were identified through hand searching. After removing duplicates, 4148 citations were screened based on title and abstract. Of these, 4013 did not meet inclusion criteria. We reviewed 135 full-text articles, of which 31 were eligible for inclusion (Fig 1). Of the 31 studies, 28 reported country-specific GBS rates from 13 LAC countries (Tables 1 & 2), 3 regional rates,[18, 35, 36] 15 background rates [18, 35–48], 11 rates during an arboviral outbreak [49–59], and 5 compared rates before and during an outbreak [27, 60–63]. Many studies presented data from Brazil (29%) and Colombia (16%), and the majority (68%) were published in 2015 or later. Of the studies that assessed background IRs, the mean number of years studied was 7.2 (range, 1–13.6 years).

**Table 1:** Characteristics of the 31 included studies of incidence of Guillain-Barré Syndrome, by location and study period.

Author/s (Year)	Country	Location	Study Period^1^	Case ascertainment	Case definition	Ages
**Central America and Mexico**						
Molinero et al (2003)	Honduras	Nationwide	1989–1999	Prospective hospital-based study of acute flaccid paralysis (AFP) cases	Asbury & Cornblath	<15
de la Peña et al (2015)	Mexico	Jalisco (state)	2005–2009	Retrospective hospital-based review of medical discharge records	Asbury & Cornblath	≥18
del Carpio Orantes et al (2018)	Mexico	North Veracruz (delegation)	2016–2017	Retrospective hospital-based review of medical discharge records	Brighton Collaboration (1–3)	All
**South America**						
Rojas et al (2009)	Argentina	Buenos Aires (city)	1999–2007	Retrospective hospital-based review of medical records	ICD-9 357.0 & NINCDS	All
Codebó et al (2016)	Argentina	Nationwide	2007–2013	Retrospective review of medical discharge records in national database	ICD-10 G61.0	All
Dias-Tosta et al (2002)	Brazil	Nationwide	1990–1996	National AFP surveillance system and medical diagnosis	Asbury & Cornblath	<15
Dourado et al (2012)	Brazil	Rio Grande do Norte (state)	1994–2007	Prospective hospital-based series	Asbury & Cornblath	All
Rocha et al (2004)	Brazil	Sao Paulo (city)	1995–2002	Retrospective review of medical discharge records	Asbury & Cornblath	All
Barcellos et al (2017)	Brazil	Northeast region	January 2008-May 2015 June-October 2015	Admission records in national hospital information system	ICD-10 G61.0	All
Souza (2018)	Brazil	Piauí (state)	2014–2016	Active hospital-based state surveillance system	Brighton Collaboration (1–3)	All
Paploski et al (2016)	Brazil	Salvador (city)	2015	Active surveillance and medical records review	Not specified	All
Nobrega (2018)	Brazil	Recife (metropolitan)	January-June, 2015	Retrospective review of medical discharge records	ICD-10 G61.0 & Brighton Collaboration (1–3)	All
Styczynski et al (2017)	Brazil	Salvador (metropolitan)	April-July 2015	Passive state surveillance system and review of medical discharge records	Brighton Collaboration (1–3)	≥12
Department of Health of Paiuí State (2016)	Brazil	Piauí (state)	November 2015-October 2016	Active hospital-based state surveillance system	Not specified	All
Rivera-Lillo et al (2016)	Chile	Nationwide	2001–2012	Passive national surveillance system	ICD-10 G61.0	All
Machado-Alba et al (2016)	Colombia	Nationwide	March 2014-September 2015 October 2015-March 2016	Private insurance diagnostic database	ICD-10 G61.0	All
Anaya et al (2017)	Colombia	Cúcuta (city)	June 2015-July 2016	Passive national surveillance system	Brighton Collaboration (1,2)	All
Tolosa et al (2017)	Colombia	Nationwide	August 2015-May 2016	Passive national surveillance system	ICD-10 G61.0	≤18
Instituto Nacional de Salud de Colombia (2016)	Colombia	Nationwide	October 2015-March 2016	Passive national surveillance system	ICD-10 G61.0	All
Salinas et al (2017)	Colombia	Barranquilla (city)	October 2015-April 2016	Passive national and local surveillance systems and medical records review	ICD-10 G61.0 & Brighton Collaboration (1–3)	All
Hart et al (1994)	Paraguay	Nationwide	1990–1991	National AFP surveillance and medical diagnosis	Asbury & Cornblath	<15
**Caribbean**						
Suryapranata et al (2016)	Aruba	Nationwide	2003–2011	Retrospective review of medical discharge records	ICD-9 357.0 & Asbury & Cornblath	All
van Koningsveld et al (2001)	Curaçao	Nationwide	1987–1999	Retrospective review of medical discharge records	ICD-9 357.0 & Asbury & Cornblath	All
Núnñez R et al (2017)	Dominican Republic	Nationwide	January 2016-October 2016	Passive national surveillance system	Brighton collaboration (1,2)	All
Balavoine et al (2017)	Guadeloupe and Martinique	Nationwide	2011–2013 2014 (chikungunya)	Retrospective review of medical discharge records	ICD-9	All
Roze et al (2017)	Martinique	Nationwide	2006–2016 2014 (chikungunya) 2016 (Zika)	Retrospective review of medical discharge records and prospective medical evaluation	ICD-10 & Brighton Collaboration (1,2)	All
Salinas et al (2017)	Puerto Rico	Nationwide	2013	Retrospective review of medical discharge records and insurance claims	ICD-9357.0 or ICD-10 G61.0 and Brighton Collaboration (1–3)	All
Dirlikov (2018)	Puerto Rico	Nationwide	2017	National surveillance system followed by medical record review	ICD-10 G61.0 & Brighton Collaboration (1–3)	All
**Regional**						
Olivé et al (1997)	7 countries^2^		1989–1991	Passive AFP regional surveillance followed by neurologist diagnosis	Asbury & Cornblath	<15
Silveira et al (1997)	4 countries^3^		1990–1994	Passive AFP regional surveillance followed by neurologist diagnosis	PAHO Polio Eradication Field Guide definition	<15
Landaverde et al (2010)	19 countries^4^		2000–2008	Passive AFP regional surveillance followed by neurologist diagnosis	PAHO Polio Eradication Field Guide definition	<15

^1^ When two dates are given, the first is before and the second during the epidemic period

^2^ El Salvador, Guatemala, Honduras, Paraguay, Peru, Mexico, and Venezuela

^3^Argentina, Brazil, Chile and Colombia

^4 ^Argentina, Brazil, Chile, Colombia, Cuba, Ecuador, El Salvador, Guatemala, Honduras, Mexico, Nicaragua, Panama, Peru, the Bahamas, Guyana, Jamaica, St Vincent and the Grenadines, Suriname, and Trinidad and Tobago

**Table 2:** Annual incidence rate of Guillain-Barré Syndrome in 31 selected studies, by location and epidemic status.

Author/s (Year)		Country	Epidemic arbovirus	Study duration (in years)	Incident cases	Mean annual cases	Population	Annual incidence rate per 100,000 persons (95% CI)^1^
**Central America and Mexico**								
Background incidence rate (IR) of GBS								
	Molinero et al (2003)	Honduras		11.0	394	36	2 627 737	1.37 (0.96–1.90)
	de la Peña et al (2015)	Mexico		5.0	45	9	4 513 718	0.20 (0.09–0.38)
IR during epidemic outbreak								
	del Carpio Orantes et al (2018)	Mexico	Zika	2.0	34	17	2 732 286	0.62 (0.36–1.00)
**South America**								
Background IR of GBS								
	Rojas et al (2009)	Argentina		9.0	26	3	145 310	2.06 (0.43–6.03)
	Codebó et al (2016)	Argentina		7.0	1 859	264	41 904 761	0.63 (0.56–0.71)
	Dias-Tosta et al (2002)	Brazil		7.0	1 678	240	52 111 801	0.46 (0.40–0.52)
	Dourado et al (2012)	Brazil		13.6	149	11	2 781 767	0.40 (0.20–0.71)
	Rocha et al (2004)	Brazil		8.0	95	12	3 000 000	0.40 (0.21–0.70)
	Barcellos et al (2017)	Brazil		7.4	2 407	325	54 711 473	0.59 (0.53–0.66)
	Rivera-Lillo et al (2016)	Chile		12.0	4 158	347	16 353 842	2.12 (1.90–2.36)
	Machado-Alba et al (2016)	Colombia		1.6	98	62	6 500 000	0.95 (0.73–1.22)
	Hart et al (1994)	Paraguay		2.0	37	19	1 747 703	1.09 (0.65–1.70)
IR during epidemic outbreak								
	Souza (2018)	Brazil	Zika	3.0	73	24	2 927 711	0.82 (0.53–1.22)
	Paploski et al (2016)	Brazil	Zika	1.0	51	51	2 920 300	1.75 (1.30–2.30)
	Nobrega (2018)	Brazil	Zika	0.5	44	88	3 890 145	2.26 (1.81–2.79)
	Styczynski et al (2017)	Brazil	Zika	0.25	48	192	3 428 571	5.60 (4.84–6.45)
	Barcellos et al (2017)	Brazil	Zika	0.42	377	905	56 445 105	1.60 (1.50–1.71)
	Department of Health of Piauí State (2016)	Brazil	Zika	1.0	23	23	3 219 257	0.71 (0.45–1.07)
	Anaya et al (2017)	Colombia	Zika	1.1	29	27	656 380	4.11 (2.71–5.98)
	Tolosa et al (2017)	Colombia	Zika	0.8	40	51	16 236 326	0.31 (0.23–0.41)
	Machado-Alba et al (2016)	Colombia	Zika	0.5	71	142	6 500 000	2.18 (1.84–2.57)
	Instituto Nacional de Salud de Colombia (2016)	Colombia	Zika	0.5	270	563	49 529 208	1.14 (1.04–1.23)
	Salinas et al (2017)	Colombia	Zika	0.5	47	93	1 218 475	7.63 (6.16–9.35)
**Caribbean**								
Background IR of GBS								
	Suryapranata et al (2016)	Aruba		9.0	36	4	100 000	4.00 (1.09–10.24)
	van Koningsveld et al (2001)	Curaçao		12.3	49	4	152 694	2.62 (0.71–6.71)
	Balavoine et al (2017)	Guadeloupe and Martinique		3.0	42	14	792 091	1.77 (0.97–2.97)
	Roze et al (2017)	Martinique		10.0	105	8	378 243	2.12 (0.91–4.17)
	Salinas et al (2017)	Puerto Rico		1.0	61	61	3 595 839	1.70 (1.30–2.18)
IR during epidemic outbreak								
	Núñez R et al (2017)	Dominican Republic	Zika	0.8	559	671	10 075 045	6.66 (6.17–7.18)
	Balavoine et al (2017)	Guadeloupe and Martinique	Chikungunya	1.0	27	27	783 336	3.45 (2.27–5.01)
	Roze et al (2017)	Martinique	Chikungunya	1.0	15	15	378 243	3.97 (2.22–6.54)
	Roze et al (2017)	Martinique	Zika	0.8	30	36	385 103	9.35 (6.55–12.94)
	Dirlikov (2018)	Puerto Rico	Zika	1.0	123	123	3 411 307	3.61 (3.00–4.30)
**Regional**								
Background IR of GBS								
	Olivé et al (1997)	7 countries		3.0	1527	509	55 934 066	0.91 (0.83–0.99)
	Silveira et al (1997)	4 countries		5.0	2 296	456	73 400 000	0.62 (0.57–0.68)
	Landaverde et al (2010)	Regional		9.0	10 486	1 165	142 086 721	0.82 (0.77–0.87)

^1^ Calculations based on annualized GBS cases and population reported in the paper or from raw data provided by authors.

### Background GBS incidence rates

**Fig 2:**
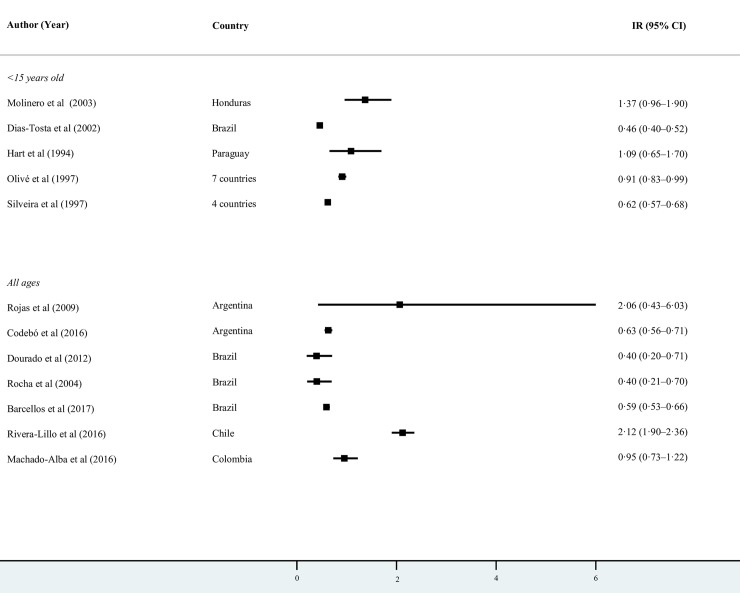
Annual background incidence rates of GBS by age in Latin America per 100,000 persons.

**Fig 3:**
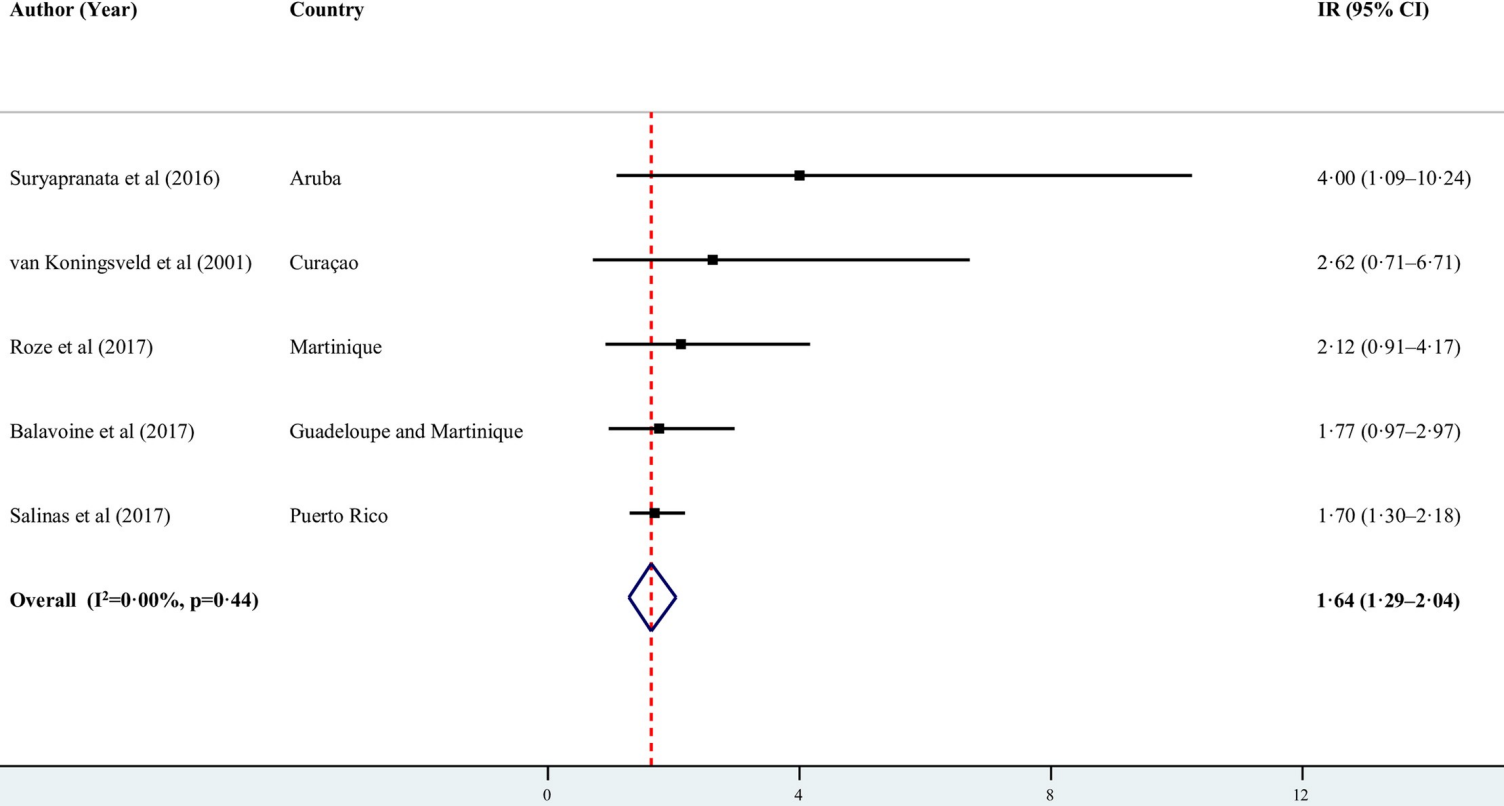
Annual and pooled background incidence rates of GBS in the Caribbean per 100,000 persons.

Among the studies in Latin America that reported background GBS IRs in all ages, the highest were reported in Chile (2.12 per 100,000; 95% CI 1.90–2.36)[44] and Argentina (2.06 per 100,000; 95% CI 0.43–6.03) [39], and the lowest in Brazil (0.40 per 100,000; 95% CI 0.20–0.71)[42] (Fig 2). High heterogeneity in Latin America and in other subgroup analyses precluded us from pooling the proportions. The pooled annual IR in the Caribbean was 1.64 per 100,000 (95% CI 1.29–2.12, *I*^*2*^ = 0.00, p = 0.44) (Fig 3).

There were large annual fluctuations in GBS IRs in any given geographic location. In Chile, over a 12-year period, IRs ranged from 1.61 per 100,000 (n = 250 cases) in 2001 to 2.35 per 100,000 (n = 402 cases) in 2010 [44]. Over a 14-year period, Dourado et al. [42] reported a range of 0.12 to 0.66 per 100,000 in Rio Grande do Norte, Brazil. Another multi-year study from Brazil estimated mean annual IRs of 0.4 per 100,000 (range, 0.3–0.6) [43]. In the Caribbean island of Aruba, over an 11-year period, Suryapranata et al. reported a range of 1 to 11 cases of GBS, the latter occurring during an outbreak of *C*. *jejuni* (IRs ranged 1.0–10.37 per 100,000). There was less fluctuation in IRs in Argentina, ranging from 0.56 per 100,000 in 2011 to 0.76 per 100,000 in 2010 over a 7-year period [40]. However, the IR of GBS among members of a private health maintenance organization in Buenos Aires averaged 1.99 per 100,000 over an 8-year period [39].

### GBS incidence rates in children

Of the studies reporting background IRs in children under 15, the lowest rates were in Brazil at 0.40 per 100,000 [18], followed by Paraguay at 0.72 per 100,000 [45], and the highest rates in El Salvador at 3.86 per 100,000 [18], Chile at 1.63 per 100,000 [18], and Honduras at 1.37 per 100,000 [37].

The age-specific distribution of GBS in the pediatric population varied widely across countries. Two multi-country studies found the highest GBS IR in the 1−4 age group: Olivé et al. reported that 47% of the GBS cases were in children 1−4 years of age [35] and Silveira et al. reported an average IR of 0.86 per 100,000 (95% CI 0.78–0.89) in this age group as compared to 0.52 per 100,000 (95% CI 0.49–0.53) among 5−14 year-olds [36]. In Paraguay, the IRs ranged from 1.7 per 100,000 among 1−4 year-olds to 0.1 per 100,000 among 10−14 year-olds [45]. In Brazil, a similar trend was reported, with 40% of the cases reported in children under 5 [41]. However, in Chile the IRs were higher among 5−9 year-old children (2.23 per 100,000) than among 0−4 year−olds (2.17 per 100,000) [44].

### Distribution of GBS by age in the population as a whole

GBS distribution by age in the general population did not follow a consistent pattern across countries. In Chile, the IR showed a bimodal distribution with a peak in the youngest ages (2.23 per 100,000 in 5−9 year-olds), and increased from 1.22 per 100,000 among 20−29 year-olds to 4.30 per 100,000 among 70−79 years-olds [44]. In São Paulo, Brazil, GBS was most common among 15−40 year-olds (0.15 per 100,000), and the IR was lowest in the over 60 (0.60 per 100,000) and under 15 (0.80 per 100,000) age groups [43]. In Rio Grande do Norte, Brazil, half of the cases were recorded among under 20 years-olds [42]. In Argentina, 37% of the GBS cases were reported in children under 14 years of age [40]. Reporting of higher IRs among children might be due to higher case detection resulting from vaccination safety and polio eradication surveillance efforts.

### Sex differences

Studies consistently found a higher burden of GBS among males. In studies of children 15 years of age and younger, the highest male-to-female ratio was documented in El Salvador (1.8:1) [35] and the lowest in Brazil (1.2:1) [41] and Honduras 1.3:1 [37]. Among all ages, the highest male-to-female ratio was documented in Aruba (2.3:1) [46], followed by Argentina (1.6:1) [40], and the lowest in Brazil (1.3:1) [42].

### GBS and arboviral infections

**Fig 4:**
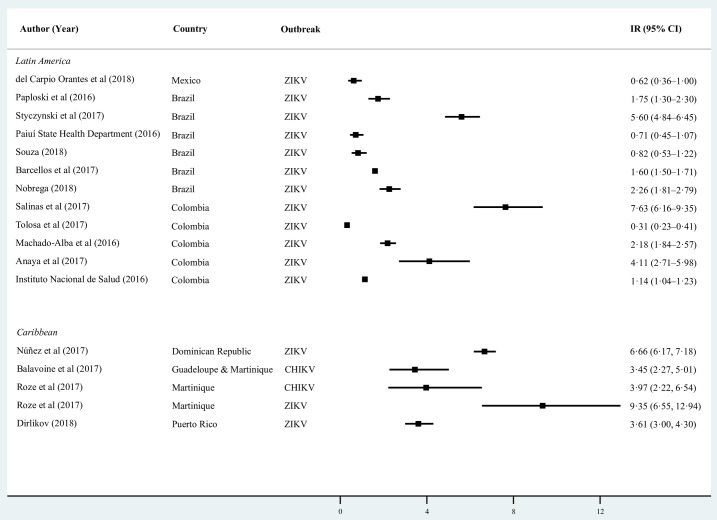
Annualized incidence rates of GBS by sub-region during arboviral epidemic outbreaks.

We found that 17 studies reported the GBS IR during arboviral disease outbreaks in seven countries; 14 during the 2015–2016 ZIKV epidemic, one during the 2014 CHIKV outbreak in the French West Indies, and 1 during DENV, CHIKV and ZIKV outbreaks in Martinique (See Fig 4). The IR in the French West Indies during the 2014 CHIKV epidemic was 3.45 per 100,000 persons [27], representing a two-fold increase from 1.77 per 100,000 during 2011–2013 (p = 0.006) [27]. The IR in Martinique increased by 4.4 times to 9.35 per 100,000 during the ZIKV epidemic from a mean annual IR of 2.12 per 100,000 during 2006–2015 [Incidence rate ratio (IRR) _(2016 vs. 2006–2015)_ = 4.52; 95% CI 2.80–7.64] [62]. In Puerto Rico, the IR increased by 2.1 times during the ZIKV epidemic from 1.7 to 3.5 per 100,000 [IRR _(2016 vs. 2012–2015)_ = 2.06; 95% CI 1.51–2.85] [48, 63].

In South America, there was substantial heterogeneity in the GBS IRs during the 2015−2016 ZIKV epidemic for the population as a whole, ranging from 7.63 per 100,000 in Barranquilla, Colombia [58], to 0.71 per 100,000 in the Brazilian state of Piauí [54]. In a multi-year study, Barcellos et al. reported a 2.7 (IRR _(2015–2016 vs. 2008–2015)_ = 2.7; 95% CI 2.38–3.07) increase in the rate of GBS hospitalizations in Brazil’s Northeast region during the peak of the ZIKV epidemic as compared to the mean rate in the eight years preceding the epidemic [60]. In Colombia, Machado et al. reported over a two-fold increase in GBS diagnoses during the peak of the ZIKV epidemic as compared to baseline rates (IRR _(2016 vs. 2014–2015)_ = 2.29; 95% CI 1.69–3.14) [61].

In Veracruz, Mexico, del Carpio Orantes reported a GBS IR of 0.62 per 100,000 during the ZIKV epidemic in 2016 [49].

We performed sub-group analyses by case-ascertainment (i.e., administrative data based on ICD codes only versus both administrative data and medical record review) to assess if the IR heterogeneity was due to this factor. We found significant within-group heterogeneity but not between-group heterogeneity, indicating that case ascertainment did not significantly bias the IRs. This held true when we performed a region-wide analysis, and when we analyzed Latin American and the Caribbean sub-regions separately (See S1 Fig).

## Discussion

The pooled background IR of GBS calculated in the Caribbean of 1.64 per 100,000 (95% CI 1.29–2.12, *I*^*2*^ = 0.00, p = 0.44) was higher than the mean IR in North America and Europe but within the estimated range for those regions (0.80−1.90 per 100,000) [13]. In LAC, background IRs of GBS ranged from 0.40 per 100,000 in Rio Grande do Norte [42] and São Paulo [43] in Brazil to 2.12 per 100,000 in Chile [44]. These background IRs exceeded the upper and lower ranges calculated by the aforementioned meta-analysis [13], but were comparable to the range reported in a 2009 GBS literature review from 0.38/100,000/year in Finland to 2.53/100,000/year in Curaçao [14].

Sejvar et al. found that the incidence of GBS increased with age [13]. However, we did not observe similar trends in the LAC population, with the GBS IR exhibiting different age-specific patterns across settings. The higher burden of GBS among males reported in this review was consistent with Sejvar et al., with an estimated relative risk for males of 1.78 (95% CI 1.36–2.33) [13].

This review synthesizes GBS incidence data from CHIKV and ZIKV epidemic outbreaks. However, data on CHIKV infection and GBS are limited to the French West Indies. The association of DENV infection with GBS is not well established. One multi-year study in Aruba found a positive correlation between number of GBS cases and laboratory-confirmed DENV infections (p = 0.004, Kendall’s tau-b) [46]. However, a study from Brazil reported no association between DENV outbreaks and GBS [64]. More data are needed on incidence of GBS during CHIKV and DENV outbreaks in the region. In terms of ZIKV, most studies showed temporal associations of ZIKV infections with GBS rates. However, fewer studies reported GBS rates at baseline and during an outbreak, in part because of sparse historical data on GBS. Dos Santos et al. reported increases in IRs during the weeks of ZIKV transmission ranging from 100% in El Salvador (95% CI 55.7–156.9) to 877% in Venezuela (95% CI 664.1–1149.6) as compared to estimated pre-ZIKV baselines [25]. Consistent with Dos Santos et al., studies that reported rates of GBS at baseline and during epidemic weeks reported increases of 106% in Puerto Rico (IRR _(2016 vs. 2012–2015)_ = 2.06; 95% CI 1.51–2.85), 352% in Martinique (IRR _(2016 vs. 2006–2015)_ = 4.52; 95% CI 2.80–7.64), 171% in Brazil’s Northeastern region (IRR _(2015–2016 vs. 2008–2015)_ = 2.7; 95% CI 2.38–3.07), and 129% in Colombia (IRR _(2016 vs. 2014–2015)_ = 2.29; 95% CI 1.69–3.14). In summary, all studies that reported rates of GBS before and during the ZIKV outbreak showed significant temporal increases in IRs during the outbreak. However, this could be due to publication bias. Furthermore, heterogeneity coupled with limited data precludes us from drawing region-wide comparisons of these differences.

ZIKV emerged in an immunologically naive population and spread rapidly throughout the Americas, with the consequent impact on human health [65]. In the upcoming years it is unlikely that the region will experience similar peaks of GBS due to ZIKV infection given the build-up of population immunity against this arbovirus [66]. However, other emerging pathogens, including different arboviruses and influenza strains [67], may trigger increases in the incidence of GBS. In the future, in addition to background GBS-triggering foodborne infections, we expect GBS incidence to be cyclical as attack rates of arboviral infections fluctuate seasonally as well as in response to population immunity. Foodborne infections are often cited as the most common GBS trigger, responsible for between 25% and 50% of GBS cases worldwide [1]. Of eight studies included in this review that examined *C*. *jejuni* as an antecedent infection to GBS, only two found a positive association. On the island of Curaçao, authors found a positive temporal association between *C*. *jejuni* infections and GBS in pre-ZIKV years (1987–1999) [47]. In Veracruz, Mexico, 75% of GBS patients tested positive for *C*. *jejuni* infection in 2017 (after the peak of ZIKV) [49]. Studies in Aruba, Brazil, and Colombia–all except the one from Aruba were conducted during the ZIKV epidemic–found no association between *C*. *jejuni* infection and GBS [43, 46, 50, 52, 55, 58]. A study in Brazil found respiratory infections to be present in 27% of GBS patients compared to 7% with gastrointestinal infections in pre-ZIKV years (1995–2002) [43]. Although examining the role of foodborne infections was outside the scope of this research, this limited evidence suggests that temporal increases in GBS incidence during ZIKV in LAC are not associated with *C*. *jejuni* infection. With more robust GBS surveillance and increased awareness, the region would be better prepared to monitor fluctuations in GBS incidence in the future. Although this review focused on GBS in endemic populations, incidence of neurological conditions in other regions might be impacted by international travelers returning from areas with ZIKV and other arboviral disease transmission, or with a sexual partner who returned from such areas [68–76].

### Strengths and limitations

Data on GBS, both historical and current, are lacking for many countries in the region. With the exception of studies using AFP data, most are recent and from ZIKV-affected countries, and baseline data are often lacking. Publication bias in favor of significant results may have limited the availability of studies that found no association between arboviral epidemics and GBS. On the other hand, ZIKV’s high public visibility most likely led to increased scientific research and publications on GBS as well as surveillance (i.e., detection) bias, particularly in countries affected by the emerging arbovirus. Few studies reported data from Central America and Mexico. Therefore, estimates for this sub-region have a high risk of bias. All but two studies reported effect sizes for the outcome of interest. However, we addressed this limitation by calculating 95% confidence intervals for all studies.

Methodological differences in case finding limit comparability across studies. The specificity and sensitivity of administrative data varies by setting [48, 77]. Rates of GBS based on administrative records or passive surveillance systems, without medical record review, may be prone to over-reporting [48] and under-reporting [35, 78], respectively. We opted to include surveillance and administrative data because of the paucity of data available. The high cost of capture-recapture methods to compare sensitivity of case identification and ascertainment methods of a rare syndrome limits the feasibility of such studies to assess GBS incidence nationwide. Several researchers have accepted the use of administrative data as a viable low-cost option to assess background GBS IRs [40, 44].

A limitation of this review is that studies employed different methodologies to ascertain GBS cases. Diagnosing GBS is complex and based on clinical observation and electrophysiological studies. Specialists who can properly diagnose GBS may be unavailable in resource-limited settings. Natural annual variations in incidence may be masked or exacerbated by imperfect case identification.

A strength of this research is that all included studies are population based. In addition, many of the studies are multi-year in duration, with a mean of 7.2 years for studies that analyzed background GBS IRs. This gives us a robust estimate of GBS incidence that balances out natural fluctuations in annual IRs. Another strength is the application of a thorough search methodology and inclusion of most languages spoken in LAC. Inclusion of ministry of health bulletins data served to balance publication bias.

Some of the included studies were not carried out with the sole purpose of measuring GBS IRs. While the studies that focused on IR calculations tended to adjust the rates based on population age structures, others did not. This introduces a source of variability. Some studies reported incidence by person-years and others as annual incidence. We addressed this issue by calculating all IRs.

Since GBS is triggered by a variety of antecedent infections, baseline incidence of GBS is critical for detecting and monitoring infectious disease outbreaks. The LAC region has been a pioneer in monitoring of GBS in children over the last 30 years [16]. Countries such as Colombia and Brazil have monitored GBS as part of DENV eradication programs [57, 79]. The ZIKV epidemic and the reported increases in GBS in the Americas have made GBS a notifiable condition in many countries. As the ZIKV epidemic has spread beyond the Americas [80–82], it is important that those countries are particularly prepared for GBS surveillance and management. Enhanced surveillance and increased research have provided us with new data to assess GBS incidence in this region. Because of its severity and lethality in the absence of adequate care, investments are needed to provide information on GBS to populations at-risk and to build healthcare providers’ capacity to diagnose GBS and follow appropriate care protocols [83, 84]. GBS poses an additional burden to health care systems, particularly in resource-limited settings.

## Supporting information

S1 File PRISMA checklist. (DOC)

S2 File MOOSE checklist. (PDF)

S3 File Detailed search strategy. (DOCX)

S1 Fig Sub-group analysis by case ascertainment. Sub-group analysis by case ascertainment: administrative data and medical record review versus ICD code only.
(PDF)

S2 Fig Risk of bias in GBS observational studies. Risk of bias in GBS observational studies.
(PDF)
