# Supplementary material for: Incidence of Guillain-Barré Syndrome (GBS) in Latin America and the Caribbean before and during the 2015–2016 Zika virus epidemic: A systematic review and meta-analysis
Source: PLoS Negl Trop Dis. 2019 Aug 26;13(8):e0007622. doi: 10.1371/journal.pntd.0007622 (PMC6730933; doi:10.1371/journal.pntd.0007622)
Supplement: S3 File — (DOCX) [file pntd.0007622.s003.docx]

**Detailed search strategy**

Qualifications of researchers:

The research team included a public health and medical librarian with expertise in systematic reviews (DLV), an epidemiologist to advise on analytical methods and conduct data validation (DCO), an arboviral disease medical expert who leads the ZikaPlan consortium (AWS), and experts in arboviral disease epidemiology (AC, YT)

Handling of articles in other languages:

All articles were screened and read in the original language as the first author is fluent in Spanish, Portuguese and English

Databases searched:

PubMed/Medline, EMBASE, Lilacs/Scielo, Web of Science Plus, Global Health (Ovid), Gideon, Population Index, CINAHL Plus and Google Scholar

Hand-searched journals:

American Journal of Tropical Medicine and Hygiene, PLOS Neglected Tropical Diseases, The Lancet, The Lancet Infectious Diseases, Emerging Infectious Diseases Journal, New England Journal of Medicine, BMJ, the Pan American Journal of Public Health, Bulletin of the World Health Organization

Ministries of health:

Argentina, Colombia, Peru, Jamaica, Puerto Rico and the Dominican Republic. These sites were selected based on a search of bibliographic references of the articles that were included for full-text review.

Full search terms:

(Guillain Barre syndrome OR Guillain Barre OR acute inflammatory polyneuropathy OR acute autoimmune neuropathy OR Landry-Guillain-Barre Syndrome OR acute inflammatory demyelinating polyneuropathy OR Miller Fisher Syndrome OR Fisher Syndrome OR acute idiopathic polyneuritis OR acute idiopathic polyneuritis) AND (((epidemiology OR epidemiologic OR epidemiological) AND (methods OR research OR surveys)) OR incidence). “Latin America and the Caribbean” were not included in the search terms to reduce the likelihood of missing relevant studies.
