# Supplementary figures and images for: Incidence of Guillain-Barré Syndrome (GBS) in Latin America and the Caribbean before and during the 2015–2016 Zika virus epidemic: A systematic review and meta-analysis
Source: PLoS Negl Trop Dis. 2019 Aug 26;13(8):e0007622. doi: 10.1371/journal.pntd.0007622 (PMC6730933; doi:10.1371/journal.pntd.0007622)

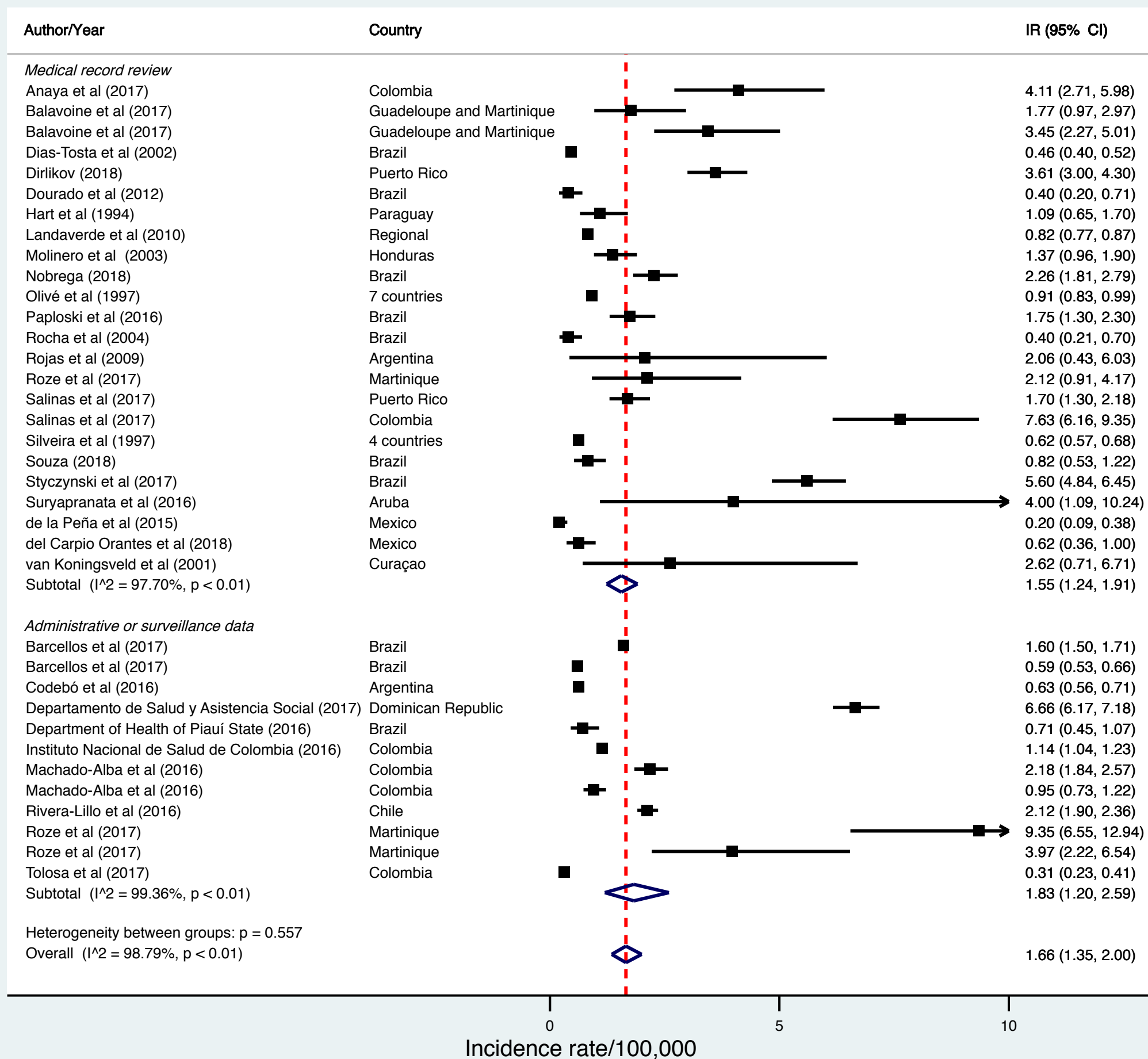

Supplement: S1 Fig — Sub-group analysis by case ascertainment: administrative data and medical record review versus ICD code only. (PDF) [file pntd.0007622.s004.pdf]
