# Supplementary material for: Incidence of Guillain-Barré Syndrome (GBS) in Latin America and the Caribbean before and during the 2015–2016 Zika virus epidemic: A systematic review and meta-analysis
Source: PLoS Negl Trop Dis. 2019 Aug 26;13(8):e0007622. doi: 10.1371/journal.pntd.0007622 (PMC6730933; doi:10.1371/journal.pntd.0007622)
Supplement: S2 Fig — Risk of bias in GBS observational studies. (PDF) [file pntd.0007622.s005.pdf]

	1. Are the methods explained in sufficient detail?	2. Was an acceptable case definition used in the study? (diagnosis certainty)	3. Was risk of case detection bias (numerator(s) for GBS incidence)) low?	4. Was the population (denominator(s) for GBS incidence) (e.g. population, hospital catchment area, etc.) clearly specified?	5. Summary item on the overall risk of study bias
Anaya et al (2017)	LOW	MEDIUM	MEDIUM	HIGH	LOW
Balavoine et al (2017)	LOW	HIGH	MEDIUM	LOW	LOW
Barcellos et al (2017)	LOW	HIGH	MEDIUM	LOW	LOW
Codebó et al (2016)	LOW	HIGH	MEDIUM	LOW	LOW
de la Peña et al (2015)	LOW	LOW	LOW	LOW	LOW
del Carpio Orantes et al (2018)	LOW	LOW	LOW	MEDIUM	LOW
Department of Health of Paiuí State (2016)	HIGH	HIGH	MEDIUM	LOW	LOW
Dias-Tosta et al (2002)	LOW	LOW	MEDIUM	LOW	LOW
Dirlikov (2018)	LOW	LOW	LOW	LOW	LOW
Dourado et al (2012)	LOW	LOW	LOW	MEDIUM	LOW
Esack (2010)	HIGH	LOW	MEDIUM	HIGH	HIGH
Hart et al (1994)	LOW	LOW	MEDIUM	LOW	LOW
Instituto Nacional de Salud Colombia (2016)	HIGH	LOW	MEDIUM	LOW	LOW
Landaverde et al (2010)	LOW	LOW	MEDIUM	LOW	LOW
Machado-Alba et al (2016)	LOW	HIGH	MEDIUM	MEDIUM	LOW
Mazia (1997)	HIGH	LOW	MEDIUM	HIGH	HIGH
Molinero et al (2003)	LOW	LOW	LOW	LOW	LOW
Nobrega (2018)	LOW	LOW	LOW	LOW	LOW
Núñez R et al (2017)	HIGH	LOW	LOW	LOW	LOW
Olivé et al (1997)	LOW	LOW	MEDIUM	LOW	LOW
Paploski et al (2016)	LOW	HIGH	MEDIUM	LOW	LOW
Ramírez-Zamora (2009)	LOW	LOW	MEDIUM	HIGH	HIGH
Rivera-Lillo et al (2016)	LOW	HIGH	MEDIUM	LOW	LOW
Rocha et al (2004)	LOW	LOW	MEDIUM	LOW	LOW
Rojas et al (2009)	HIGH	HIGH	MEDIUM	MEDIUM	LOW
Roze et al (2017)	LOW	LOW	LOW	LOW	LOW
Salinas, Major et al (2017)	LOW	LOW	LOW	LOW	LOW
Salinas, Walteros et al (2017)	LOW	LOW	LOW	LOW	LOW
Santos (2009)	HIGH	HIGH	HIGH	HIGH	HIGH
Silveira et al (1997)	LOW	LOW	MEDIUM	LOW	LOW
Souza (2018)	LOW	LOW	MEDIUM	LOW	LOW
Styczynski et al (2017)	LOW	LOW	LOW	LOW	LOW
Suryapranata et al (2016)	LOW	LOW	LOW	LOW	LOW
Tolosa et al (2017)	LOW	HIGH	MEDIUM	LOW	LOW
van Koningsveld et al (2001)	LOW	LOW	LOW	LOW	LOW
Webster-Kerr (2016)	HIGH	LOW	HIGH	LOW	HIGH

Risk of bias:

- LOW
- MEDIUM
- HIGH
